# Differences in Cognitive-Motor Interference in Older Adults While Walking and Performing a Visual-Verbal Stroop Task

**DOI:** 10.3389/fnagi.2018.00426

**Published:** 2019-01-09

**Authors:** Bettina Wollesen, Claudia Voelcker-Rehage

**Affiliations:** ^1^Department of Human Science, Faculty of Psychology and Movement Science, University of Hamburg, Hamburg, Germany; ^2^Sports Psychology, Institute of Human Movement Science and Health, Faculty of Behavioral and Social Sciences, Chemnitz University of Technology, Chemnitz, Germany

**Keywords:** aging, dual task performance, walking, cognition, physical functioning, concerns of falling, mental health

## Abstract

**Objectives**: Studies using the dual-task (DT) paradigm to explain age-related performance decline due to cognitive-motor interference (CMI) which causes DT costs (DTCs) revealed contradictory results for performances under DT conditions. This cross-sectional study analyzed whether differences in demographics, physical functioning, concerns of falling (CoF), and other mental factors can explain positive and negative DTCs in older adults while walking in DT situations.

**Methodology**: *N* = 222 participants (57–89 years) performed a single task (ST) and a DT walking condition (visual-verbal Stroop task) in randomized order on a treadmill. Gait parameters (step length, step width) were measured at a constant self-selected walking speed. Demographics [age, Mini Mental Status Examination (MMSE)], physical functioning (hand grip strength), CoF [Falls Efficacy Scale International (FES-I)], and mental factors [Short Form-12 (SF-12)] were assessed. An analysis of variance (ANOVA) was used to reveal subgroup differences. A four-step hierarchical regression analysis was conducted to identify which variables determine the DTC.

**Results**: Three subgroups were identified: (1) participants (*n* = 53) with positive DTCs (improvements under DT conditions); (2) participants with negative DTCs (*n* = 60) in all gait parameters; and (3) participants (*n* = 109) who revealed non-uniform DTCs. Baseline characteristics between the subgroups showed differences in age (*F*_(2,215)_ = 4.953; *p* = 0.008; *η*^2^ = 0.044). The regression analysis revealed that physical functioning was associated with positive DTC and CoF with negative DTC.

**Conclusion**: The results confirmed a huge inter-individual variability in older adults. They lead us to suggest that factors causing performance differences in DTCs needs to be reassessed. Functional age seems to determine DTCs rather than calendric age. Psychological variables particularly seem to negatively influence DT performance.

## Introduction

Age is associated with sensorimotor change and changes in the musculoskeletal system. In combination or interaction, these age-related changes lead to decrements of locomotor coordination, and they also have an impact on walking performance by decreasing gait stability. Behavioral data, as revealed by the use of biomechanical measurements, showed that effects on the locomotor system are expressed in reduced step length (Scott et al., 2015), gait speed (Verghese et al., 2009; Morrison et al., 2016), as well as increased double support time (Maki, 1997; Verghese et al., 2009; Scott et al., 2015), step length variability (Maki, 1997; Verghese et al., 2009), step width (Maki, 1997), or stumbling (Berg et al., 1997). Moreover, older people have problems adapting their walking abilities at higher gait speeds (e.g., to catch a bus) or while walking over uneven surfaces (Berg et al., 1997). All of these aspects can be described as external perturbations that have a negative impact on postural control and therefore decrease gait stability (for additional definition see the review by van Emmerik et al., 2016) and cause an increased risk of falling (Hausdorff et al., 2001).

In daily situations, the locomotor system needs to integrate sensory information and to coordinate movements according to the situation. Gait performance also depends on sensorimotor and cognitive functions. It is proposed that with increasing age, sensory and motor aspects of walking performance increasingly require cognitive control, attention, and supervision. However, age is associated with reduced cognitive processing efficiency (e.g., decrease in nerve conduction speed, increased lateralization; Hedden and Gabrieli, 2004) and in turn, a decrease in cognitive performance, such as diminished response time, working memory, and processing of multiple tasks. These age-related cognitive changes might affect daily task performance (Stawski et al., 2006). In this context, more and more studies indicate a correlation between age-related declines in the sensory and motor system, as well as in cognitive functioning (Li and Lindenberger, 2002).

The cognitive processing of locomotion in dual- or multi-tasking situations is measured to identify people’s susceptibility to adopt impaired gait patterns, often resulting in an increased risk of falling (e.g., crossing a street while observing traffic flow; Faulkner et al., 2007). Adding a secondary motor or cognitive task typically reduces gait stability due to interference during information processing (Wollesen et al., 2016) measured as reduced movement accuracy and movement coordination (Al-Yahya et al., 2011). An age-related reduction in cognitive performance or cognitive-motor interference (CMI) affects how older people cope with such dual-task (DT) situations in daily life.

### Cognitive-Motor Interference (CMI) During Dual-Task Walking in Older Adults

Walking in our natural environment can be considered a DT scenario that requires increasing cognitive resources with increasing age. The level to which walking performance is affected by CMI is typically expressed as the DT cost (DTC). DTCs are calculated as the percentage of decrements in performance of a DT relative to the performance of a single task (ST). Age-related declines of performance whilst walking in DT situations have been extensively investigated (Li et al., 2001; Hollman et al., 2007; Bock, 2008). For instance, an age-related decline in gait performance has been observed when conducting arithmetic, memory, or visual tasks concurrently to walking (Lindenberger et al., 2000; Beurskens and Bock, 2012). These performance declines in DT walking situations have been considered in light of several theoretical positions (see Wollesen et al., 2017a for an overview). Recent systematic reviews on empirical findings and theoretical models (Lacour et al., 2008; Wollesen et al., 2017a) showed that CMI rises with increasing task complexity of the motor and/or cognitive task and according to individual abilities and resources (Lacour et al., 2008). Moreover, the task domain (stimulus-response mode) was found to be a critical moderator variable (Riby et al., 2004). Hence, task settings including controlled processes (e.g., inhibiting information) or motor components (e.g., carrying a tray) showed more decrements in DT performance in older adults than other task combinations. Moreover, studies indicate that increasing difficulty levels (from DT to multi-task-performance or with different task complexities, e.g., from processing speed to executive tasks) also increase the effects of DT on gait decrements (Hall et al., 2011; Venema et al., 2013; Li et al., 2014; Menant et al., 2014; MacLean et al., 2017). However, in contrast to previous research, a study by Plummer-D’Amato et al. (2012) failed to show effects of different cognitive loads on DT walking performance. They only found an effect for different walking conditions (comfortable vs. fast vs. obstacle crossing). Further, older adults often reveal higher DTC than young adults (Lindenberger et al., 2000; Beurskens and Bock, 2012). Some studies reported inconsistent results (Muir-Hunter and Wittwer, 2016) or even less DTC in older than in younger adults for DT walking conditions, where the cognitive tasks did not require visual attention (e.g., walking with a spelling task; Bock, 2008).

It still remains unclear which individual factors or resources might explain DTCs or decrements in daily situations that require the management of different simultaneously performed tasks when walking speed remains constant. Several factors have been discussed that might influence CMI of older adults. Possible influencing factors include complexity of the motor and/or cognitive task and the task domain, age-related motor or cognitive declines (Muhaidat et al., 2013), task prioritization (posture first hypothesis), and previous falls or concerns of falling (CoF; Ambrose et al., 2013).

The* Task Prioritization Model* (Yogev-Seligmann et al., 2010) accounts for the individuals’ strategies used during DT performance. It proposes that older adults prioritize motor performance, if the motor task may induce loss of balance (Brown and Bennett, 2002; Chapman and Hollands, 2007). This prioritization is used to compensate CMI and to reorganize the cognitive-motor resources (Li and Lindenberger, 2002) or to reduce the risk of falling. Yogev-Seligmann et al. (2012) found that older adults with adequate balance abilities and capacity to identify hazards are able to focus on cognitive performance as long as balance is maintained. This result was discussed as older adults prioritizing walking over memorizing to protect themselves from falls, a view known as “posture first hypothesis” (Shumway-Cook and Woollacott, 2000; Schaefer and Schumacher, 2011; see Li et al., 2013; for discussion of mixed results).

Being a *faller* has also been shown to influence gait performance, such as step width and step length (e.g., Barak et al., 2006; Lindemann et al., 2008; Nordin et al., 2010; Kirkwood et al., 2011), as well as DTC. Fallers are often not able to shift attention to the motor task in DT situations (Schaefer and Schumacher, 2011). Furthermore, the combination of high-risk task settings (e.g., elevated surface) and a secondary task also leads to problems of task prioritization in healthy older adults (Schaefer et al., 2015).

Nevertheless, there is some evidence that older adults with a reduced postural reserve (motor abilities to maintain balance) have more decrements of gait performance regardless of their cognitive performance in ST and DT situations (Holtzer et al., 2014). Most of the studies focusing on fall prevention report higher decrements of gait parameters in fallers, including gait speed, step length, step width, and double support time (Maki, 1997; Beauchet et al., 2009; Muhaidat et al., 2013). These changes apply especially in situations that require adapting a faster gait speed (Barak et al., 2006). Declines are associated with an increased risk of falling (Beauchet et al., 2009; Menant et al., 2014). Furthermore, fallers have poorer motor precondition (e.g., reduced physical fitness or muscle strength; Freire Júnior et al., 2017). Additionally, studies have reported that older adults at risk of falling had poorer mobility judgment in a virtual reality DT walking situation (crossing a street while listening to music or writing messages) and therefore experienced more collisions with oncoming cars (Nagamatsu et al., 2011; Neider et al., 2011). Recent studies added findings showing that impaired executive functioning and attention impact the walking performance of older fallers (MacAulay et al., 2015; Cornu et al., 2016).

Another explanation for DTC of older adults are CoF. Older adults with higher levels of CoF have difficulties to inhibit or ignore irrelevant information from the environment in the process of balance control (Young and Mark Williams, 2015). Therefore, during the cognitive process of movement coordination, the CoF seems to compete for the limited resources of attentional focus to maintain balance control (Young and Mark Williams, 2015), resulting in instability and fall risk. For ST walking performance, a meta-analysis by Ayoubi et al. (2015) revealed significant effects of CoF expressed in increased gait variability. Under DT conditions, Donoghue et al. (2016) found reduced gait speed and step length, especially for older persons who reduced their daily physical activity due to their CoF. Therefore, CoF appears to have an impact on mental processes and might reduce the available resources for task managing in DT situations.

The mental status also seems to play a role. For example, older people with depressive disorders showed reduced DT performance (Nebes et al., 2001). Older adults with unipolar depressive disorders have shown problems inhibiting information, and they also have greater response times in comparison to healthy control groups in DT situations (Gohier et al., 2009). Moreover, Hausdorff et al. (2008) found a correlation between mental well-being and DTC in older adults.

In addition, muscle strength or physical functioning, expressed by reduced hand grip strength (Rantanen et al., 1999; Bohannon, 2008), for example, might influence the DT performance of cognitive-motor DT situations. Reduced hand grip strength has been shown to be an indicator of frailty (Rantanen et al., 1999; Bohannon, 2008), muscle strength, mortality, quality of life, and/or heart health (Norman et al., 2011). In this vein, Guedes et al. (2014) revealed an interaction of frailty (assessed as reduced hand grip strength) and reduced DT performance while walking. Therefore, one might assume that the functional condition can free up cognitive capacity for motor coordination which would otherwise be needed to compensate impaired motor functioning.

In summary, recent literature allows us to derive different explanations for DTCs or decrements. They might be a result of: (1) age-related motor or cognitive declines in general; (2) of task difficulty of the cognitive task or the stimulus-response mode of the cognitive task, especially of tasks that need executive control; (3) the complexity of the motor task (walking situation); (4) task prioritization (posture first); (5) previous falls or CoF; or (6) of mental; or (7) physical functioning, or a combination of several factors.

Nevertheless, extensive research about CMI in older adults has not sufficiently discussed older individuals’ preconditions, such as physical functioning (e.g., hand grip strength), psychological factors (e.g., CoF), or mental state (i.e., mental well-being) and the resulting positive or negative DTC. Therefore, the aims of this study were: (1) to identify whether DTC of older adults were positive or negative when performing a visual-verbal Stroop task while maintain walking speed; and (2) to analyze the individuals’ preconditions (age, physical functioning, CoF) that might have an impact on positive or negative DTC. We hypothesized that older participants can be clearly classified into groups with and without DTC during DT walking (for step length and step width) based on individual characteristics such as age and CoF.

## Materials and Methods

This study consists of a secondary analysis of all baseline data from participants who took part in a larger study program to develop DT managing training. The program was approved by a local Ethics Committee of the Chamber of Physicians (PV4376).

### Participants

Overall, a total sample size of *N* = 240 participants (mean age and SD: 72.35 ± 5.4 years, age range 57–89, *n* = 177 female, *n* = 63 male) was recruited for the study program. Recruitment was conducted using advertisements in local newspapers. The inclusion criteria were: independent living, age 65–85 years, and the ability and mobility [Short Physical Performance Battery (SPPB) > 9; ability to walk without walking aids] to join the study program. Exclusion criteria were: acute or chronic disease with documented influence on balance control (e.g., Parkinson’s Disease or Diabetes), use of gait assistance (e.g., walking canes, frames, rolling walkers), a Mini Mental Status Examination (MMSE; Folstein et al., 1975) of less than 25 points indicating any cognitive impairment, and color blindness. A total of 18 participants were excluded (*n* = 14 due to an SPBB score <9, *n* = 3 due to an MMSE <25, and *n* = 1 due to age). All participants were informed about the study goals and risks and signed informed consent prior to any testing according to the Declaration of Helsinki. There was no financial compensation for participating in the study.

All included participants completed a standardized questionnaire assessing demographics, anthropometric data, and comorbidities. Health-related quality of life was examined using the Short Form-12 questionnaire (SF-12 Bullinger and Kirchberger, 1998; see Table 1). The analysis includes a physical and mental SF-12 score.

**Table 1:** Mean (SD) or number (%) of the groups for the demographic characteristics of *N* = 222 participants at baseline.

Characteristics	Negative DTC (1) (*n* = 60)	Positive DTC (2) (*n* = 53)	Positive and negative DTC (3) (*n* = 109)
Age (year)	70.56 (4.7)*	73.50 (5.7)	72.43 (5.0)
Females, number (%)	75.0	75.9	72.1
Height (cm) females	164.5 (7.8)	164.1 (6.2)	164.7 (6.5)
Height (cm) males	177.2 (9.0)	178.9 (4.9)	176.6 (7.2)
Weight (kg) females	69.4 (12.2)	66.2 (11.3)	69.2 (11.4)
Weight (kg) males	83.4 (12.4)	84.8 (8.1)	84.0 (11.2)
SPPB (score out of 12)	11.43 (0.9)	11.22 (0.8)	11.24 (0.9)
Walking speed (km/h)	3.19 (1.0)	3.13 (0.7)	3.21 (0.6)
Hand grip force (kg)	22.9 (10.8)	19.7 (7.8)	20.3 (10.1)
MMSE (>25)	27.8 (2.5)	26.7 (2.4)	27.7 (3.0)
FES-I (score out of 64)	21.2 (4.3)	20.0 (3.7)	19.7 (3.0)
SF-12 physical (reference score age group 37.76 ± 12.27)	47.1 (8.5)	45.9 (9.2)	49.3 (8.2)
SF-12 mental (reference score age group 50.24 ± 10.81)	50.0 (8.2)	52.0 (7.5)	51.4 (7.3)
Right answers Stroop test sitting	27.6 (3.0)	26.4 (4.5)	27.2 (3.1)
Right answers Stroop test walking	27.5 (2.6)	26.9 (3.2)	27.4 (3.3)

BMI, Body Mass Index; SPPB, Short Physical Performance Battery; FES-I, Falls Efficacy Scale International; SF-12, Short Form-12 questionnaire; MMSE, Mini Mental Status Examination. *p < 0.05.

### Outcome Measures

#### Treadmill Walking

Subjects performed a 30-s walking test at a self-selected constant speed on an h/p/cosmos motorized treadmill with integrated sensors to measure peak plantar pressure and other gait kinematics (Zebris, Isny, Germany).

Self-selected walking speed was determined *via* a staircase method, which means walking up to a certain level of comfortable speed and increasing and decreasing speed until a comfortable pace was achieved (range between 0.7 km/h up to 6.0 km/h). Gait data were collected for both feet at 100 Hz. Standardized measurements of gait kinematics (step length, step width) were conducted with the included FDM-T software: each trial had a duration of 30 s.

Before the test sessions started, all subjects practiced treadmill walking. With familiarization periods of about 5 min, participants were allowed to practice until they felt comfortable with the training device (see Wollesen et al., 2017a). Self-selected gait speed was constantly used for the ST and DT conditions. Participants were secured by a safety harness.

#### Cognitive Task

Subjects performed 30-s visual-verbal Stroop tests with 16 events of congruent and incongruent color words (e.g., the word “blue” presented in yellow letters). The colors red, blue, yellow, and green were used. Participants had to name the color of the font in which the letters were presented but not the actual word spelled by the letters. The time interval between word insertions varied between 0.8 ms and 1.2 ms to avoid rhythm of occurrence. The tests differed in the sequences of word colors.

To avoid a learning effect, we conducted three different versions of the Stroop test, where congruent and incongruent stimuli were presented *via* a computer screen in randomized order. All Stroop tests were recorded on video presentation within the software (Garage Band; Apple; Cupertino, CA, USA). The video included the verbal response of the participants to the observed color word on the screen. The number of correct answers was monitored, recorded, and analyzed. The analysis was based on all stimuli, irrespective of the congruency of the stimuli (e.g., the word “red” was presented in blue color and the participant answered blue or the word was “red” and was presented in red and the participant answered red).

#### Condition Cognitive Performance (Sitting and Walking)

In the ST (sitting) and DT (walking) condition, subjects performed the visual-verbal Stroop test with 16 events of color words (written in blue, red, green, yellow). In sitting condition stimuli were projected onto a white wall 2 m in front of the participants (for further details see Wollesen et al., 2016).

#### DT Condition Walking

In the DT walking condition, subjects performed the visual-verbal Stroop as described above while walking on the treadmill. The words were displayed in the size of 40 to 58 cm × 20 cm at a distance of 415 cm. The trial lasted 30 s and its length was matched with the length of the walking sequence. Participants were not introduced to strategies for prioritizing their gait patterns or the cognitive task.

#### Concerns of Falling (CoF)

The German version of the Falls Efficacy Scale International (FES-I, Dias et al., 2006) was used to examine concerns about falling during 16 daily activities. The 16 items are rated as “not at all concerned” (1) to “very concerned” (4). All items were summed up to a FES-I score. Higher scores are indicative of greater CoF (Delbaere et al., 2010).

#### Physical Functioning

The maximum hand grip strength was measured (Bohannon, 2008) using a Jamar^®^ Hydraulic hand dynamometer (Model 5030J1, J. A. Preston Corporation, Clifton, NJ, USA) as a predictor of physical functioning. The hand dynamometer was adjusted to the individual’s hand size. Participants were asked for their dominant hand (left or right). Each hand was tested twice with a 1-min rest between trials. The test took place in a standing position with arms extended perpendicular to the body. The maximum value of the two trials for the dominant hand served as the result.

### Data Analysis

Addressing the changes in walking performance under DT conditions, the data analysis focused on the DTC for motor performance while walking. Following Doumas et al. (2009), DTCs were calculated using the formula: (ST-DT/ST) *100. DTCs were calculated for the walking parameters (step length and step width).

Based on their DTC, participants were separated into three groups:

1. Persons who showed decreased gait performance under DT conditions, indicated by an increased step width and reduced step length = negative DT performer;
2. Persons who increased their gait performance under DT conditions, indicated by a reduced step width and increased step length = positive DT performer;
3. Participants who showed non-uniform adaptions to the DT condition, such as decreased step width and decreased step length or increases in both parameters = non-uniform DT performer.

All statistical analyses were performed using SPSS 24 computer software (IBM statistics Armonk, NY, USA). To analyze differences between the three groups of older adults (negative, positive, non-uniform performer), analysis of variance (ANOVA) were calculated for all DTC outcome parameters (DTC of step length and step width). Significance was set at *α* = 0.05; normal distribution was tested *via* the Kolmogorov-Smirnov test. Effect size is presented as partial eta square ($\eta_{\text{p}}^{2}$; small effect $\eta_{\text{p}}^{2}$ ≥ 0.08, moderate effect $\eta_{\text{p}}^{2}$ ≥ 0.20, and $\eta_{\text{p}}^{2}$ ≥ 0.32 large effect). A Bonferroni correction was applied for all *post hoc* comparisons.

Furthermore, we analyzed potential influencing factors on DTC. Therefore, Pearson product-moment correlations were computed using all cognitive (right answers for Stroop task performance while sitting and walking) and psychological variables (SF-12 mental score, FES-I-scores), physical characteristics (gait speed, physical functioning, SF-12 physical score), and relevant demographics (age) of the participants. Next, a four-step hierarchical regression analysis was conducted to identify which variables determine the positive, negative, or non-uniform DTC while walking.

In the first step, age, and in the second step all relevant physical characteristics (hand grip strength, SF-12 physical score, and preferred gait speed) were included. In the third step, the psychological components were entered (SF-12 mental score, FES-I-scores). In step 4, the model was adjusted to cognitive DT performance (right answers sitting and walking).

## Results

Table 1 describes the physical characteristics and demographic conditions of the participants (*N* = 222).

The only significant group difference observed in Table 1 was the age of the subjects. Participants with positive DTC were older than the two other groups (*F*_(2,215)_ = 4.953; *p* = 0.008; $\eta_{\text{p}}^{2}$ = 0.044).

The range of positive and negative DTC for step width and step length was between 1% up to 95% for step width, and 1% up to 60% for step length.

Table 2 shows the correlations between DTC and the physical, cognitive, and demographic characteristics of the participants.

**Table 2:** Correlations between the different outcome variables.

	1	2	3	4	5	6	7	8
1 Age								
2 Hand grip strength	0.034						
3 SF-12 phys	−0.127	−0.175						
4 SF-12 men	0.148	0.114	−0.078					
5 FES-I	**−0.144**	−0.207**	−0.273**	−0.365**				
6 Right answers sitting	−0.317**	0.090	0.074	−0.073	−0.076			
7 Right answers walking	−0.286**	0.059	0.014	0.047	−0.145	0.655**		
8 Gait speed (km/h)	−0.023	0.414**	−0.001	0.069	−0.193**	0.055	−0.013	
9 DTC	0.123	−0.108	−0.001	0.095	−0.186*	−0.022	0.000	0.017

SF-12 phys men, Score of SF 12 Questionnaire; DTC, dual task costs; FES-I, Falls Efficacy Scale International; *p < 0.05, **p < 0.01. Bold values highlight significant differences. Phys, physical score; Men, mental score.

There were some significant correlations between the participants’ physical and cognitive conditions. The scores of the FES-I were correlated with hand grip strength (see Table 2); participants with higher hand grip strength had reduced FES-I scores. In addition, a higher physical and mental well-being was associated with lower FES-I scores. Gait speed was positively correlated with hand grip strength and was reduced with increasing FES-I scores.

The differences in the examined gait variables for the three subgroups of DT performance are documented in Table 3.

**Table 3:** Comparison of the walking parameters of the different DTC groups.

Gait variable		Negative DTC (1) (*n* = 60)	Positive DTC (2) (*n* = 53)	Non-uniform (*n* = 109)	Group differences	
					*F*	*P*
						$\eta_{\text{p}}^{2}$
**Single-Task**						
Step width [cm]		11.1 (3.4)	12.5 (3.8)	11.8 (3.6)	2.065	0.129
						0.019
Step length [cm]	l	48.8 (13.9)	43.5 (9.7)*	46.1 (9.0)	3.396	**0.035**
						0.030
	r	49.0 (13.8)	43.4 (10.0)*	46.2 (9.2)	3.755	**0.025**
						0.033
**Dual Task condition**						
Step width [cm]		13.3 (3.7)	10.8 (3.7)*	11.3 (3.7)	7.495	**0.001**
						0.064
Step length [cm]	l	45.6 (14.1)	46.7 (9.8)	46.7 (9.3)	0.208	0.813
						0.002
	r	46.1 (14.1)	46.9 (10.2)	47.0 (9.6)	0.124	0.883
						0.001
**Dual Task costs**						
Step width [%]		23.5 (32.2)*	−14.1 (11.9)	−2.6 (19.4)	44.002	**0.000**
						**0.287**
Step length [%]	l	−6.1 (9.5)*	8.4 (11.1)	1.3 (8.2)	34.230	**0.000**
						**0.238**
	r	−6.2 (10.4)*	9.1 (11.5)	1.9 (9.2)	32.561	**0.000**
						**0.229**

*Significant *post hoc* test of group comparisons; l, left foot; r, right foot. Bold values highlight significant differences.

Regression analysis of relevant physical, cognitive, and psychological characteristics and demographic conditions of the participants and DTC is shown in Table 4.

**Table 4:** Summary of hierarchical regression analysis for variables predicting motor DTC (positive or negative or non-uniforn DTC) as dependent variable.

	Step 1		Step 2		Step 3		Step 4	
	*B*	*β*	*B*	*β*	*B*	*β*	*B*	*β*
1. Age	−0.002	−0.13	−0.006	−0.40	−0.015	−0.098	−0.026	−0.165
2. Hand grip strength,			−0.025	−0.246	−0.037	−0.359**	−0.038	−0.372**
SF-12 phys			0.009	0.088	0.009	0.082	0.008	0.076
Gait speed			0.019	0.022	0.009	0.011	0.023	0.026
3. SF-12 men,					0.006	0.052	0.005	0.045
FES-I					−0.080	−0.339*	−0.081	−0.345*
4. Right answers sitting							−0.071	−0.262
Right answers walking							0.038	0.149
*R^2^*	0.013		0.249		0.413**		0.444	
Δ*R2*			0.062		0.109		0.026	

Step 1: Age Step 2: Hand grip strength. SF-12 phys, gait speed (km/h), Step 3: SF-12 men, FES-I and Step 4: Right answers sitting and right answers walking, *p < 0.05, **p < 0.01.

**Figure 1:**
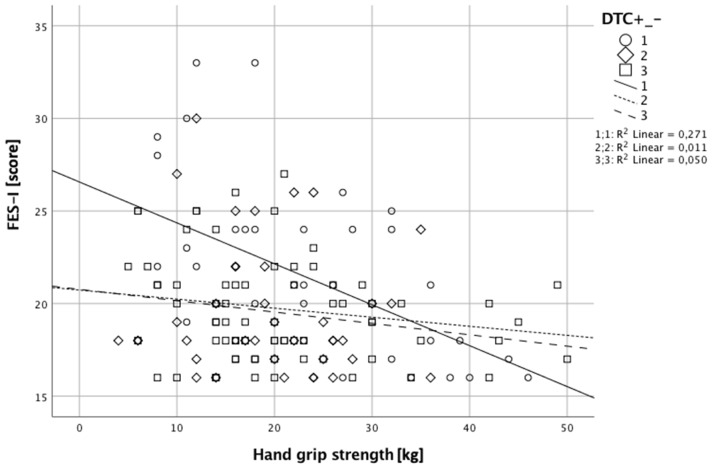
This image shows the interaction of Falls Efficacy Scale International (FES-I) and hand grip strength. Negative dual task (DT) performers showed higher concerns of falling (CoF) and greater hand grip strength. Positive DT performers had reduced hand grip strength and less CoF. Scores of FES-I and a hand grip strength for non-uniform performers were between the scores of the two other groups.

Steps 1 and 2 of the regression analysis of age and the physical parameters did not indicate a significant effect. In step 3, mental well-being and FES-I were integrated into the model. The overall model was significant (*F*_(6,75)_ = 2.575; *p* = 0.025; see Table 4). In this step, significant effects for hand grip strength (*p* = 0.007) and FES-I (*p* = 0.003) were observed. Participants with negative DTC showed higher CoF. Participants with negative DTC had higher hand grip strength (see Figure 1; Table 4).

The analysis of step 4 included the cognitive performance in the Stroop test. The significant overall effect remained (*F*_(8, 73)_ = 2.234; *p* = 0.034), as well as the significant effects for hand grip strength (*p* = 0.005) and FES-I (*p* = 0.003; see Figure 1).

## Discussion

**Figure 2:**
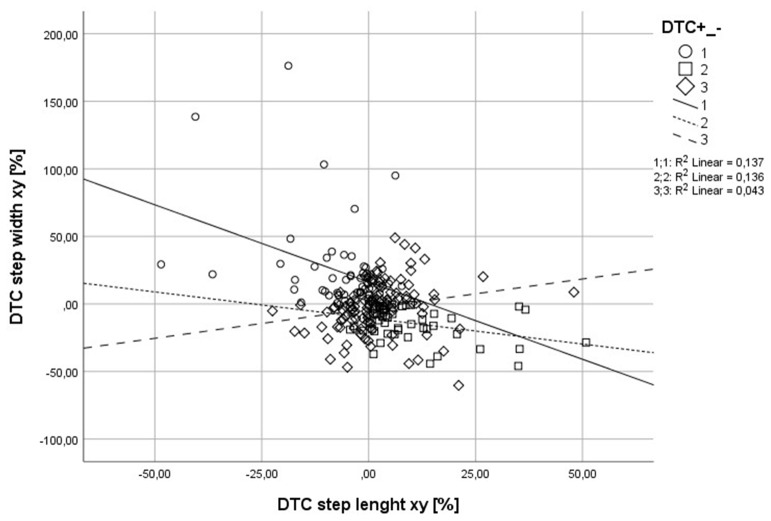
This image shows that participants with negative DT costs (DTCs) had greater decrements for step width and step length than the other two groups, whereas participants with positive DTC revealed improved step length with reduced step width. The non-uniform participants were between the two other groups.

Motor-cognitive DTC during walking in older adults might be a result of age-related motor, cognitive declines, previous falls, or CoF. However, previous research revealed heterogeneous results and did not sufficiently discuss whether other individuals’ preconditions, like physical functioning, psychological factors (CoF), or mental factors, might affect DTC positively or negatively. Therefore, the aims of this study were: (1) to identify whether DTC of older adults were positive or negative when performing a visual-verbal Stroop task while walking; and (2) to analyze the individuals’ different preconditions that might have an impact on positive or negative DTC. Our main hypothesis was that participants could be clearly classified into two groups revealing either positive or negative influence of the secondary task on walking performance (step length and step width). Overall, we were able to classify three groups with different DTC patterns: (1) participants with positive DTC, which means their step length increased and step width decreased (positive DT performer); (2) participants with negative DTC expressed by reduced step length and increased step width (negative DT performer); and (3) participants that either improved or reduced only one of the gait parameters (non-uniform DT performer) (Figure 2). With respect to demographic characteristics, the groups only differed in age. Specifically, the positive DTC group was older than the other two groups. Moreover, physical functioning and CoF might be associated with DTC, as well.

### Positive, Negative, and Non-uniform DTC

We were only able to classify 50 percent of the participants into the groups with overall positive or negative adaptions to the DT situation, which was unexpected. The other 50 percent showed either positive or negative effects on step width or step length, meaning step width and step length increased or vice versa, thus revealing non-uniform DTC. These opposed changes in the gait parameters might be strategies to compensate the additional cognitive load to secure gait performance (Beurskens and Bock, 2012; Wrightson et al., 2016). Thus, results indicate that performance does not necessarily decline under DT conditions as long as there is room for compensation. As the majority of earlier studies focused on one gait parameter only (mostly gait speed) and did not control for different DT performance levels, they might have misinterpreted the negative DTC when analyzing the gait decrements. In our study, gait speed was assessed in the first session to determine comfortable walking speed and then remained constant across the whole trial (motor driven treadmill). Thus, our participants did not reveal declines in gait speed. Nevertheless, all participants revealed performance changes under DTC conditions in at least one gait parameter (step length or width), but more than two thirds revealed either decline or compensation. There were also participants who showed only one or two percent variance between ST and DT performance or even positive DTC. This is why we suggest that DTC of older adults performing cognitive-motor tasks such as walking are not negative in general, but depend of the type of measurement or might be a compensation strategy (Li et al., 2001; Bock, 2008). The observed gait adaptions to the CMI of all participants might be a result of a compensation process due to the increased cognitive load. It has been suggested that these adaptions are the individual’s compensation strategies to increased task complexity (Hausdorff et al., 2001; Schaefer and Schumacher, 2011; Wollesen et al., 2016). In addition, it needs to be reflected that there is still a lack of information about the degree to which a certain change in step width and step length might be a positive or a negative adaption to an increased cognitive load while walking. Moreover, the walking parameters that should be observed are not clearly identified or described by existing studies [absolute values of gait kinematics, like the step length and step width, or measurements of variability, e.g., as expressed by Auvinet et al. (2017) or Hausdorff et al. (2008)]. We only analyzed the absolute values of the measurements, as their might be an error propagation if additional calculations were added to the standard measurements. Previous research of our measurement setup showed poor interclass correlation coefficients (ICC’s) for gait variability outcome variables (Wollesen et al., 2017a).

In contrast to standard measurements of cognitive performance, like reaction times, the complex coordination of walking performance cannot be described with only one variable. However, a clear explanation as to which walking variability will be effected most by CMI cannot yet be answered by the existing literature.

Furthermore, with respect to age, we found unexpected group differences in the participants with positive and negative DTC. Participants with performance decrements under DT conditions were younger than participants with positive DTC. These results contradict the findings of Plummer-D’Amato et al. (2012), who hypothesized that there is an overall age-related decrement on DT performance. In addition, it remains unclear why age did not correlate with walking speed as reported by e.g., Donoghue et al. (2016). Our results confirm the idea that age is not the only variable to explain DTC. Individual characteristics, termed as inter-individual variability (see for example Baltes et al., 1999), might have a greater impact on CMI than age itself. On the other hand, it needs to be considered that the age difference between the two groups of positive and negative DT performers was only 3 years. The results might have differed, if there had been a difference of 10 years or more.

### Potential Indicators of DTC

As revealed by the regression analyses, DTC were associated with physical functioning (grip strength) and psychological factors (CoF). Contradictory to our expectations, participants with positive DTC were older and revealed lower physical functioning (reduced hand grip strength). Reduced physical functioning along with higher age has been described as a potential factor for negative DT performance in previous literature (Beurskens and Bock, 2012). Our findings confirm the idea of physical decline with aging, but we found improved walking performance under an increased cognitive load in this group. Therefore, other individual preconditions besides age, like physical or cognitive functioning, also seem to matter.

The observed reduced hand grip strength as one parameter of reduced physical fitness or frailty (Bohannon, 2008; Rantanen et al., 1999) was associated with positive DTC when performing an executive function task while walking. Thus, it might indicate that, next to strength, additional motor preconditions are required to perform and maintain motor performance under more challenging requirements, such as DT conditions. This relationship was also reported by Voelcker-Rehage et al. (2010), who found that physical fitness indexed by muscular strength was related to cognitive performance. However, this idea was not supported by our results.

Another unexpected finding was that, for the older and less physically fit participants, the additional cognitive load benefitted movement coordination during DT walking, as shown by reduced DTC. Comparable results have been found for tasks like cuing for patients with Parkinson’s disease (Lim et al., 2005) and could be explained by the Supra postural task model (Stoffregen et al., 1999, 2000, 2007; Swan et al., 2004). Following the Supra postural task model, in contrast to the “posture first hypothesis”, the secondary cognitive task is the main movement goal and balance performance is organized to fulfill the goal (Stoffregen et al., 1999, 2000). Following this idea, the DT situation becomes the new focus of attention and replaces dysfunctional motor-coordination or executive aspects. The participants are highly concentrated on cognitive performance and motor performance improves. However, the data of this study cannot give a clear explanation of this phenomenon. Additional research comparing participants with positive and negative DTC is needed to gain insights into the mechanisms of resource allocation of older adults.

Since there were no group differences in cognitive performance of the Stroop task, the presented results indicate that all participants used the same strategy. Participants maintained a high level of correct answers during the Stoop task under DTC conditions [90% of correct answers in comparison to 80% correct answers revealed by van Iersel et al. (2008)], indicating that they focused on cognitive performance (as shown in previous studies, see Wollesen et al., 2017a,b) and did not use a “gait first” strategy. Hence, the participants in our study did not act according to the “posture first hypothesis” as expected by the task prioritization model (Hausdorff et al., 2001). These findings are in line with other studies that also failed to confirm the “posture first hypothesis” (e.g., Li et al., 2012; Janouch et al., 2018). The study by Janouch et al. (2018) used a street crossing task in a virtual reality setting with increasing task complexities, while the study by Li et al. (2012) focused on treadmill walking with two different task complexities of an arithmetic task. Since the two studies, as well as our study, used a laboratory setting, the deviating results might be explained by the unreal conditions (virtual reality, treadmill): they could have had an impact on task prioritization, because the participants might have felt secure in the laboratory environment. On the other hand, one could argue that the self-selected gait speed of less than 1 m/s was a security mechanism, which already addressed the situation on the treadmill under the ST condition. Moreover, participants with CoF adopted the additional load mainly by an increased step width to increase the base of support. In contrast to participants without CoF, this might be a posture first mechanism. However, it remains unclear whether this can be specified as a conscious decision by the participants to secure gait performance.

In comparison to the other groups, CoF was higher in participants with negative DTC, and CoF were significantly associated with DTC. Earlier studies also found gait decrements for persons with higher CoF (Rochat et al., 2010; Donoghue et al., 2016; del-Río-Valeiras et al., 2016). Our results confirmed the findings that CoF has (besides physical functioning) the highest impact on walking performance in DT situations. According to the review by Young and Mark Williams (2015), CoF lead to difficulties inhibiting irrelevant information and, together with the cognitive task, this information needs resources of the working memory. All of the resources compete for the attentional focus which is needed for movement control. Following this, fear or CoF might have the same effect as a DT itself (Young and Mark Williams, 2015), and participants with high CoF may have fewer resources available for performing the task itself in comparison to participants with less CoF, and therefore show more gait decrements in DT situations.

Nevertheless, the analysis of the presented DT gait data showed that CMI while walking does not generally occur. Moreover, the question is why we identified such a great number of participants who have positive gait changes in DT situations. Our regression model suggests that a good functional and psychological state, here expressed as grip strength and fewer CoF, might be factors influencing motor performance under demanding DT conditions. Besides the different models that explain CMI in older adults, considering (individual) influencing factors and a broader approach to explain DTC in different task complexities is needed.

## Limitations

One limitation of this study was that we did not control for cognitive DTC, e.g., reaction times. Assessing the cognitive DTC might give more insights about the adaption processes of the different DTC performers. This aspect needs to be addressed in future studies. However, we controlled cognitive performance by counting the correct answers for the Stroop task.

Moreover, the measurement setup addressed changes of the gait parameters while maintaining gait speed under the ST and DT conditions. According to the literature, the participants might have reduced their walking speed from ST to DT, which was not possible under the conditions of this study.

In addition, participants with CoF should be asked if the treadmill condition increases or reduces their concerns, and what kind of safety strategies they use, if they are afraid of falling.

## Conclusion

Our results indicate that individual preconditions should be considered when calculating DTC and when deriving conclusions for appropriate training programs. Similarly, neuroimaging studies found that imagined walking involves more cognitive control and less automated processing in low- compared to well-functioning adults (Godde and Voelcker-Rehage, 2010) and that ST gait training reduces this cognitive involvement, particularly in low-functioning persons (Godde and Voelcker-Rehage, 2017). This leads to the conclusion that we need to control these parameters in our future research projects more carefully. We particularly recommend controlling the physical fitness and CoF as standardized instruments to describe the participants’ characteristics for DT studies. Future DT studies should consider inter-individual differences in DTC when developing and evaluating training approaches or fall prevention programs.

## Author Contributions

BW conducted the study idea and the experimental design was developed by BW and CV-R. The data analysis was done by BW and CV-R. The manuscript was written by BW and added by CV-R.

## Conflict of Interest Statement

The authors declare that the research was conducted in the absence of any commercial or financial relationships that could be construed as a potential conflict of interest.

## Abbreviations

ANOVA: analysis of variance
CoF: concerns of falling
CMI: cognitive-motor interference
DT: dual-task
DTC: dual-task costs
MMSE: Mini Mental Status Examination
SPPB: Short Physical Performance Battery
ST: single task.
